# Internal Derangement of the Knee-Joint

**Published:** 1856-08

**Authors:** Gurdon Buck

**Affiliations:** Attending Surgeon to the New York Hospital


*Internal Derangement of the Knee-Joint.* By Gurdon Buck, M. D. Attending Surgeon to the New York Hospital.

Under the above title the late William Hey, of Leeds, in his "Practical Observations on Surgery, &c." (London, 1814), has described an ailment affecting the knee-joint suddenly, and unaccompanied by any external marks which would disclose its nature. The following case corresponds with great exactness to Mr. H.'s description; and, inasmuch, as it is one of rare occurrence, and the diagnosis of which is perplexing, a report of it may not be unacceptable to the columns of the *Times.*

On the morning of May 24th, 1856, Mr. S., of Lowell, Mass., called on account of a lameness of his left knee that had suddenly occurred the evening before, at the moment of rising up from a position in which his knee was sharply flexed under him. He could now no longer apply the sole of his foot to the floor in walking, but was obliged to bear his weight on his toes, with the knee maintained in a slightly flexed position, and as if ankylosed. Any attempt to straighten the knee to its full extent was resisted on account of the pain it produced, which was referred to the anterior space between the inner condyle of the femur and the corresponding head of the tibia. At this point *alone*, pressure gave pain when applied with the end of the thumb forced deep into the space between the bones. It was thought, too, that the end of the thumb did not penetrate as deep into this space as in the other knee. There was no tenderness on pressure of any other part of the joint. There was no swelling or other change in the form of the joint. The same accident had occurred to him on several previous occasions; but Mr. S. had been able to get prompt relief without surgical aid, by having the limb stretched and repeatedly flexed and extended. In this instance, however, his efforts had been unsuccessful.

Regarding a displacement of the inner semi-lunar cartilage forwards, as the cause of this derangement of the joint, the reduction of it was attempted in the following manner, as advised by Mr. Hey: The patient being placed in a chair, the limb was raised above a horizontal position and the leg flexed on the thigh to its full extent, and then extend in like manner, at the same time pressure being applied over the advanced cartilage with the end of the thumb. This latter movement of extension and pressure gave severe pain, while flexion caused none. This manœuvre was repeated several times, till, at length, while urging the extension to its full degree, the patient suddenly grasped the knee with both hands and begged we would proceed gradually, as he felt the cartilage slowly slipping back to its place. In another moment he sprang upon the floor, and stamping with his lame foot, exclaimed with delight, "I am all right again!"—*New York Med. Times.*

